# Editorial: Techniques Advances and Clinical Applications in Fused EEG-fNIRS

**DOI:** 10.3389/fnhum.2019.00408

**Published:** 2019-11-22

**Authors:** Zhen Yuan, Xin Zhang, Mingzhou Ding

**Affiliations:** ^1^Faculty of Health Sciences, University of Macau, Macau, China; ^2^Cenre for Cogntive and Brain Sciences, University of Macau, Macau, China; ^3^National Laboratory of Pattern Recognition, Institute of Automation, Chinese Academy of Sciences, Beijing, China; ^4^Department of Biomedical Engineering, University of Florida, Gainesville, FL, United States

**Keywords:** fNIRS (functional near infrared spectroscopy), EEG and ERP, multimodal responses, cognition, brain disorders

Electroencephalography (EEG) and functional near-infrared spectroscopy (fNIRS) are two very important non-invasive neuroimaging techniques, which provide us a unique opportunity to unveil where and when neural information processing is taking place in human brain. EEG recordings capture the summation of postsynaptic potentials of thousands and millions of pyramidal neurons while fNIRS data denote the hemoglobin changes induced by firing neurons. More importantly, the hemodynamic response detected by fNIRS can only indirectly quantify the neural activity, whereas EEG can offer temporally fine and direct measure of neural activity.

Interestingly, EEG-fNIRS fusion data can offer us unique perspectives on brain activation and connectivity. Consequently, the combination of these two neuroimaging technologies is able to enhance each's performance and compensate for each's disadvantage. Besides, there is a potential to combine fNIRS and EEG to inspect the neurovascular coupling mechanism, which can give complementary information about the functioning of the brain. Neurovascular coupling refers to the relationship between local neural activity and subsequent changes in hemodynamic responses, where brain activation is always accompanied by a complex sequence of cellular, metabolic, and vascular processes.

Specifically, this Research Topic aims to utilize combined EEG and fNIRS techniques to examine the relationship between hemodynamic signals in the frontal cortex and various ERP components of the whole brain. It is expected that the combined neural features are able to reveal the complex neurovascular coupling mechanism underlying various cognitive tasks, brain intervention, and brain disorders. It is also expected that the combined ERP and fNIRS signals were best able to differentiate the patients and healthy controls with improved detection accuracy.

Altogether eight articles were published for this special issue, which involves various perspectives of cognition, behavior and brain disorders, such as individual differences, brain-computer interactions (BCI), neurorehabilitation, and clinical implications.

By combing EEG and fNIRS together, brain activity and connectivity of several cognitive functions were explored. For instance, individual differences in math ability and its effect on brain activation when solving hard and easy math questions were inspected (Artemenko et al.). Additionally, the neural mechanisms of action observation were examined, in which the neural decoding was accessed in detail by this pilot study (Ge et al.). Further, the role of the inferior parietal lobule associated with a reaching task was carefully demonstrated (Zama et al.).

The applications in brain-computer interfaces (BCI) by using fused EEG-fNIRS were as well-illustrated by the interesting work form this special issue (Khan et al.), in which the hemodynamic responses with an accuracy of 86% can be predicated based on the modified vector phase diagram and the power of EEG signals (Khan et al.). Moreover, a brief review was carried out to demonstrate the present state and future directions of EEG-fNIRS in robot-assisted gait rehabilitation (Berger et al.).

The clinical applications of concurrent EEG-fNIRS recordings were also addressed for this special issue. For example, one important study was performed, indicating that integrated EEG-fNIRS indicator can be considered as a valid means to examine the efficacy of clinical treatment, such as neurofeedback training in schizophrenia patients (Balconi et al.). In addition, it is suggested that the fNIRS-EEG feature can serve as an objective measure of pain perception (Peng et al.).

Besides the EEG-fNIRS fusion studies, there is one more study exploring how sub-threshold rTMS can modulate brain cortical excitability and connectivity by recording concurrent TMS-fNIRS data (Li et al.).

In summary, this special issue supports an essential role of fused EEG and fNIRS in contributing to human brain science with different latencies and activation regions. More importantly, the multimodal neuroimaging technique paves a new avenue for improving the understanding of neural mechanism underlying various cognitive tasks and brain disorders.

## Author Contributions

All authors listed have made a substantial, direct and intellectual contribution to the work, and approved it for publication.

### Conflict of Interest

The authors declare that the research was conducted in the absence of any commercial or financial relationships that could be construed as a potential conflict of interest.

